# Feasibility of NAD(P)/NAD(P)H as redox agents in enzymatic plasmonic gold nanostar assays for galactose quantification

**DOI:** 10.1098/rsos.230825

**Published:** 2023-10-11

**Authors:** Tozivepi Aaron Munyayi, Danielle Wingrove Mulder, Engela Helena Conradie, Barend Christiaan Vorster

**Affiliations:** Centre For Human Metabolomics, Department of Biochemistry, North West University Potchefstroom, 11 Hoffman Street, Potchefstroom 2531, South Africa

**Keywords:** gold nanostars, biosensor, immobilized, biorecognition, bioassay, redox agents

## Abstract

Plasmonic colorimetric sensors have emerged as powerful analytical tools in biochemistry due to their localized surface plasmon resonance extinction in the visible range. Here, we describe the feasibility of NAD(P)/NAD(P)H as redox agents in enzymatic plasmonic gold nanostar (AuNS) assays for galactose quantification using three model enzymes, GalDH, AR and GalOx, immobilized separately on polyvinylpyrrolidone-capped AuNS scaffolds. These highly specific, sensitive and selective bioassays induce the transformation of AuNS into quasi-spherical nanoparticles during the biorecognition of galactose in water and synthetic blood matrices. As a result, using our inexpensive and simple AuNS plasmon bioassays, the presence of galactose may be detected spectrophotometrically and by the naked eye.

## 1. Introduction

Nicotinamide adenine dinucleotide (NAD+), phosphorylated nicotinamide adenine dinucleotide (NADP+) and their respective reduced forms, NAD(P)H, are charge carriers in numerous cellular reactions [1,2]. NAD(P)/NAD(P)H redox couples are ubiquitous cofactors used by many oxidoreductase enzymes and their nicotinamide region is responsible for reversible redox processes [3]. Oxidoreductases are a family of enzymes that catalyse redox reactions and the shuttle of electrons from a donor (reductant) to an acceptor (oxidant) molecule in the presence of cofactors [4]. The nicotinamide moiety of NAD(P)H absorbs light at 340 ± 30 nm and emits blue fluorescence at 460 ± 50 nm. By contrast, the corresponding oxidized forms NAD(P)+ are not fluorescent and absorb at 260 nm [5,6]. NAD(P)/NAD(P)H redox couples, which are by-products of oxidoreductase enzymes, are essential biomarkers in analytical biochemistry for the direct or indirect measurement of diverse substrates [7]. Thus, assessing the participation of the NAD(P)/NAD(P)H redox couples in metabolism may aid in diagnosing pathological disorders *in vitro* and *in vivo*.

In 1957, NADH's first fluorescence spectrophotometric detection was published [8]. Beutler devised an endogenous fluorescence-based approach in 1964, recognizing that NAD(P)H might be used as an endpoint signal in galactose screening protocols [9–12]. This technical advancement paved the way for using NAD(P)H to reduce/oxidize dyes to monitor enzyme(s) activity, resulting in colorimetric, spectrophotometric and fluorescence assays [13–15].

Subsequently, simple micro-fluorimetric assays were developed with the potential to simultaneously quantify galactose and galactose-l-phosphate using a cascade of enzymes [16–18]. Other techniques including high-performance liquid chromatography (HPLC), bacterial micro-assay, spectrophotometry and tandem mass spectrometry (MS/MS) have also been used for galactose concentration quantification with more sensitivity and precision than enzyme-based methods [19–21]. However, these methods and techniques require expertise and are costly; hence, they are unfit for rapid screening in resource-constrained areas.

The Leloir pathway is the premier route for galactose metabolism [22–24]. However, in case of failure, auxiliary pathways can be activated involving distinct enzymes [25]. In auxiliary galactose metabolism pathways, galactose dehydrogenase (GalDH; EC 1.1.1.48) catalyses the dehydrogenation reaction of galactose in the presence of NAD+, producing d-galactose-1,5-lactone and NADH [26]. Aldose reductase (AR; EC 1.1.1.21) is a cytosolic NAPH-dependent enzyme that catalyses the reduction of galactose to galactitol and NADP+ [27]. Galactose oxidase (GalOx; EC 1.1.3.9) is a metalloenzyme that catalyses the oxidation of galactose to galacto-hexodialdose and hydrogen peroxide with strict C-6 primary hydroxyl group regioselectivity [26,28]. Their biochemical properties such as efficiency, specificity and good biodegradability make them fit for rapid enzymatic determination of blood galactose [29,30].

During the last decade, significant development of oxidoreductase-based diagnostic test kits and innovative biosensor designs has been witnessed for various analytical and clinical applications [31,32]. Recently, gold nanoparticles such as gold nanostars (AuNS) have been used as signal transducers and scaffolds in enzymatic-based colorimetric assays [33]. These methods are usually based on tunable localized surface plasmon resonance (LSPR) and colloidal stability in the absence or presence of an analyte resulting in different colours [34]. Extensive research has been published on electrochemical NAD(P)/NAD(P)H biosensors functionalized with varying materials of nanocomposite and oxidoreductase enzymes [35–40]. To the best of our knowledge, limited research outputs have been reported for the colorimetric detection of NAD(P)/NAD(P)H using plasmonic gold nanoparticles immobilized with hydrogenase or reductase enzymes.

Here, we describe the feasibility of NAD(P)/NAD(P)H as redox agents in enzymatic plasmonic gold nanostar assays for galactose quantification. Three model enzymes, GalDH, AR, and GalOx, were immobilized separately on PVP-capped AuNS scaffolds. Upon the biorecognition of galactose, the AuNS will act as a signal transducer concomitantly producing rapid and quantitative colorimetric signals detectable spectrophotometrically and by the naked eye. This study contributes to the feasibility of using NAD(P)/NAD(P)H as redox agents in the development of simpler, faster, and less expensive colorimetric assays suitable for resource-constrained areas.

## 2. Material and methods

### 2.1. Reagents and materials

The following reagents were used to synthesize, functionalize and conjugate gold nanostars: gold(III) chloride hydrate (HAuCl_4_·*x*H_2_O, 99.9%), silver nitrate (AgNO_3_, 99.9%), HEPES (C_8_H_18_N_2_O_4_S, 99.5%), sodium chloride (NaCl, 99.0%), polyvinylpyrrolidone (PVP 10 000 (C_6_H_9_NO)*_n_*, 99.0%), galactose (C_6_H_12_O_6_, 99.0%), glucose (C_6_H_12_O_6_, 99.0%), 3,3′-dithiobis(sulfosuccinimidyl propionate) (DTSSP, C_14_H_14_N_2_O_14_S_4_Na_2_), sodium hydroxide (NaOH, 98.0%), hydrogen peroxide (H_2_O_2_, 30% w/w), Tris buffer (NH_2_C(CH_2_OH)_3_, 99.7%), galactose dehydrogenase (GalDH, approx. 80 U mg^−1^ protein, 95.0%), aldose reductase (AR, 1 mg ml^−1^, 95.0%), galactose oxidase (GalOx, 3000 U g^−1^, 98.0%), reduced nicotinamide adenine dinucleotide phosphate (NADPH, C_21_H_26_N_7_Na_4_O_17_P_3_·*x*H_2_O, 97.0%), reduced nicotinamide adenine dinucleotide (NAD, C_21_H_27_N_7_Na_2_O_14_P_2_·*x*H_2_O, 97%) and nicotinamide adenine dinucleotide hydrate (NADH, C_21_H_27_N_7_O_14_P_2_·*x*H_2_O, 97.0%) were purchased from Sigma-Aldrich (Johannesburg, South Africa) and were used without any purification. Supplemented fetal bovine serum (Ham's F-12K) was purchased from Thermo Fisher Scientific (Johannesburg, South Africa). Blank urine was purchased from Industrial Analytical (Johannesburg, South Africa). Lastly, ultrapure water (ddH_2_O, 18.2 MΩ cm**^−^**^1^) was used for preparing all solutions from the high-purity chemicals and reagents**.**

## 3. Characterization

### 3.1. Instrumentation

All spectra for the research were obtained using an HT Synergy microplate spectrophotometer scanned at 400–900 nm using Gen5.1 software (BioTEK, Agilent Technologies, Santa Clara, CA, USA). The morphology of the functionalized nanostars was determined with high-resolution transmission electron microscopy (HR-TEM, Tecnai F20, JEOL, Freising, Germany) at an acceleration voltage of 200 kV. HR-TEM images were captured using air-dried spotted copper grids (Agar Scientific). ImageJ software (University of Wisconsin at Madison, Madison, WI, USA) was used to measure average particle core diameter and arm counts on a sample size of 100 particles. The chemical compositions of the samples were determined qualitatively using scanning electron microscopy and energy-dispersive X-ray spectroscopy (SEM-EDS, Bruker, Billerica, MA, USA). The elemental concentrations in the samples were quantified using inductively coupled plasma mass spectrometry (ICP-MS, Spectro AMETEK, Inc, Germany). SEM-EDS and ICP-MS were used to quantify and confirm the chemical makeup of the samples precisely. ICP-MS was used to quantify elements at trace levels in the samples. NMR was performed at 500 MHz on a Bruker Avance III HD NMR spectrometer (Bruker, Billerica, MA, USA) to illustrate the presence of the capping agent and enzyme(s) after AuNS functionalization. The characterization of the functionalized AuNS was determined using a Vacutec electrophoresis gel apparatus (Vacutec, Johannesburg, South Africa). The electrophoresis was done using 0.5% agarose and 0.5× Tris borate EDTA buffer (TBE buffer) at pH 8. The samples were prepared using 20 µl AuNS mixed with 4 µl of 80% glycerol and run at 40 V for 45 min. 0.25% (w/v) coomassie blue staining for 4 h with subsequent destaining with (90% w/v isopropanol + 10% w/v glacial acetic acid) was used to ascertain successful bioconjugation as seen by the protein bands on the gels. The gels were kept in ddH_2_O (Millipore, 18.2 ΩM) for quality imaging using a Bio-Rad imaging system (Bio-Rad Laboratories, CA, USA). NMR and gel electrophoresis demonstrated that the capping and enzyme(s) were present after functionalization and attached to the samples.

### 3.2. Preparation of gold nanostars

PVP-capped AuNS were synthesized using the one-pot HEPES and silver-mediated protocol as proposed in our previous work with minor tailoring [41]. Succinctly, 2 ml of 100 mM HEPES buffer (pH 7.4) was added to 3 ml deionized water (Millipore, 18.2 ΩM), followed by 20 µl of 50 mM gold(III) chloride trihydrate (HAuCl_4_·*x*H_2_O) and 4 µl of 1 mM silver nitrate (AgNO_3_). The screw cap tubes were rotator mixed for 5 min and incubated at room temperature for 25 min until the solution turned blue. The nanostars were then capped with 600 µl of 2.5 mM PVP, and the tubes were rotator mixed for 5 min and incubated for 1 h at room temperature. The capped AuNS sample suspension was cleaned up twice by centrifugation for 35 min at 2170*g* and was resuspended in 500 µl distilled water.

### 3.3. Suitability of NAD, NADPH and H_2_O_2_ for redox-mediated change in gold nanostar morphology

The initial phase of the assays assessed the capacity of NAD+, NADPH and H_2_O_2_ to induce redox-mediated colorimetric and UV–visible spectral changes to PVP-capped AuNS. This phase was evaluated using a redox potential-based model. The reagents were pipette mixed in water to ensure a final volume of 200 µl in the following strict order for optimum results: 20 µl of 10 mM Tris buffer (pH 8.4) followed by the addition of 15 µl AuNS-PVP. In each assay, 5 µl increments until 20 µl of 50 mM H_2_O_2_ and 8 mM NAD+/NAD(P)H were pipette mixed, respectively. Finally, the assays were incubated for 5 min at room temperature before adding the detection solution (2 µl of 10 mM AgNO_3_ + 20 µl of 150 mM NaOH), and UV–visible spectral measurements were taken immediately.

### 3.4. Preparation of gold nanostar bioconjugates

The capped AuNS sample suspension was cleaned up twice by centrifugation for 35 min at 2170*g* and was resuspended in 500 µl of 100 mM HEPES (pH 6.9) after removal of the supernatant. 100 µl of 5 mM freshly made up DTSSP compatibilizer was added to three sets of 2 ml resuspended capped AuNS and the tubes were rotator mixed for 5 min and incubated for 30 min at room temperature. Following the incubation step, 100 µl of 8 mM NAD/NADPH was complexed with 150 µl of 2 mg ml^−1^ GalDH/AR, respectively, and 150 µl of 0.5 mg ml^−1^ GalOx was added to the DTSSP functionalized AuNS (in each tube respectfully) and left to incubate at 4°C for 2 h for enzyme and cofactor conjugation and immobilization [42]. The bioconjugates were cleaned up twice by centrifugation for 35 min at 2170*g* and were resuspended in 500 µl ddH_2_O (Millipore, 18.2 ΩM) and stored at 4°C.

### 3.5. Gold nanostar bioconjugate stability assay

The stability of AuNS bioconjugates was assessed in blank urine, supplemented cell culture medium and serum flocculation assays at room temperature for 3 h. Spectral readouts for a 1 : 1 solution of matrices and AuNS bioconjugates were captured and analysed every 5 min for 3 h. Because the AuNS bioconjugates are used as biosensors, their stability in matrices for longer periods of time is less important because typical assay formats' incubation time is less than 3 h. Overall, two assessments are made here: (1) storage stability in a water matrix at 4°C and room temperature for 96 h and (2) short-term stability during the analytical procedure in different matrices for 3 h. A blue UV–visible spectral shift and a shortening of the spectral curve, as shown by the loss and shift of the AuNS bioconjugate's longitudinal peak, were regarded as indicative of instability [43].

### 3.6. Gold nanostar bioconjugate colorimetric assay

In this phase, the ability of biocatalytically produced NAD(P)/NAD(P)H and H_2_O_2_ to induce redox-mediated colorimetric and UV–visible spectrum modifications in AuNS bioconjugates was evaluated. To achieve the best results, the reagents were pipette mixed in water to a final volume of 200 µl in the following specific order: 15 µl of 10 mM Tris buffer (pH 8.4) was added, followed by 20 µl AuNS bioconjugate. In each assay, 0–30 µl of 2 mM galactose was added in 5 µl increments, followed by a 15 min incubation at 37°C. Finally, the detection solution (2 µl of 10 mM AgNO_3_ + 20 µl of 150 mM NaOH) was added to the assays, followed by a 1–5 min incubation at 37°C before spectral reading measurement. Different incubation durations following the addition of the detection solution demonstrate how quickly the assay generates plausible colorimetric readings.

The final phase of the assays assessed the efficacy of the bioconjugated AuNS in colorimetric signal generation in a synthetic blood matrix. The synthetic blood matrix constituted a 1 : 1000 (water : synthetic blood) ratio spiked with 2 mM galactose and 8 mM NAD+/NAD(P)H for AuNS-PVP-GalDH and AuNS-PVP-AR bioconjugates. For the AuNS-PVP-GalOx bioconjugate, the coenzymes were excluded.

### 3.7. Gold nanostar bioconjugate specificity assay

The efficacy of the AuNS bioconjugates was investigated through analyte specificity assays, for which galactose was replaced by its epimer glucose. 2 mM glucose solution was used for the analyte specificity tests. The assays were optimized and pipette mixed chronologically as the galactose assays. Finally, as for the other assays described above, UV–visible spectral measurements were acquired.

## 4. Results

In the present study, HEPES-mediated PVP-capped AuNS were synthesized. The synthesized nanoparticles were characterized and used in NAD(P)+/NAD(P)H and H_2_O_2_ coupled redox-mediated colorimetric assays.

**Figure 1.**
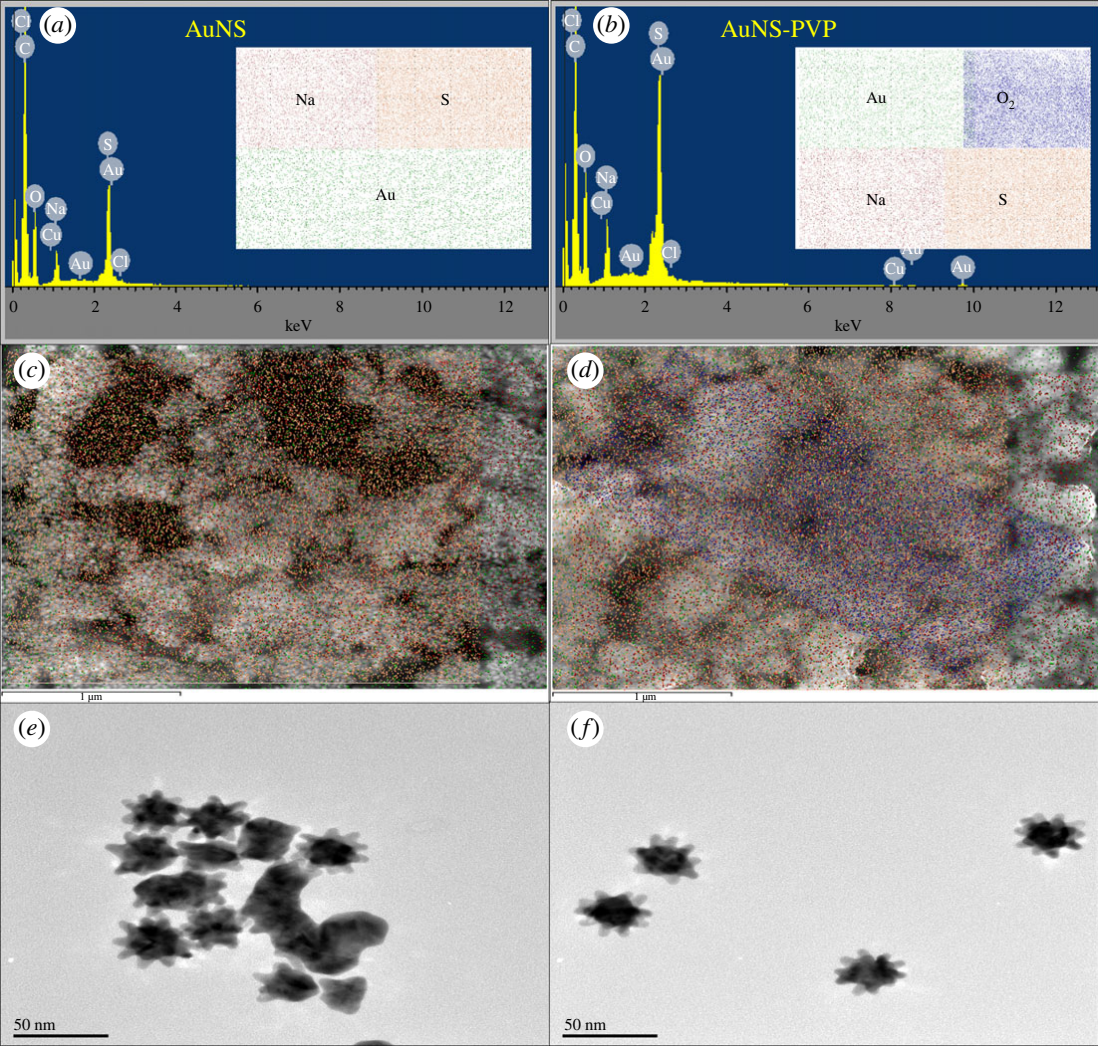
EDS spectra, SEM-EDS elemental mapping profiles, and HR-TEM images for AuNS. (*a*,*c*,*e*) Uncapped AuNS; (*b*,*d*,*f*) PVP-capped AuNS.

Figure 1 shows the elemental composition, mapping, and HR-TEM images of AuNS. The AuNS were mainly composed of gold, sulfur and carbon and were moderately monodispersed with a 10-armed morphology and a diameter of approximately 40–42 nm.

**Figure 2.**
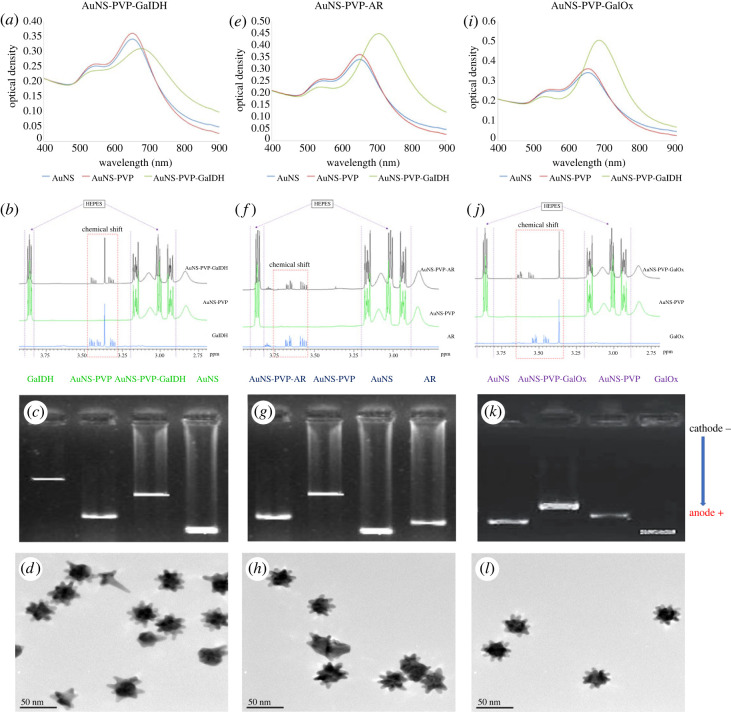
UV–visible spectra profiles, ^1^H-NMR spectra profiles, electrophoretic profiles and HR-TEM images for AuNS bioconjugates. (*a–d*) AuNS-PVP-GalDH, (*e–h*) AuNS-PVP-AR, (*i–l*) AuNS-PVP-GalOx.

UV–visible and ^1^H-NMR spectral profiles, along with electrophoresis and HR-TEM images of GalDH, AR and GalOx AuNS bioconjugates, are presented in figure 2. Two distinct absorption peaks, corresponding to the longitudinal (approx. 690 nm) and transverse plasmon resonance modes (approx. 540 nm), are observed in the UV–visible spectra (figure 2*a*,*e*,*i*) [44].

The ^1^H-NMR spectra confirm the presence of the respective enzymes after bioconjugation (figure 2*b*,*f*,*j*). Complementarily, the electrophoretic migration patterns suggest successful capping and enzyme bioconjugation to the AuNS and not its mere presence in the sample (figure 2*c*,*g*,*k*). HR-TEM images in figure 2*d*,*h*,*l* show that AuNS maintain their shape after conjugation while exhibiting slightly different dispersions.

**Figure 3.**
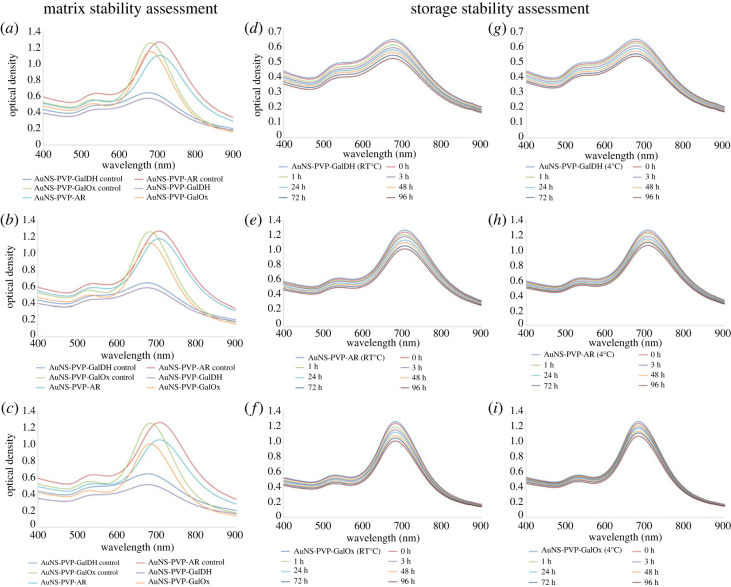
UV–visible spectra of AuNS bioconjugate stability after 3 h incubation in (*a*) blank urine; (*b*) serum; (*c*) supplemented cell culture medium. Short-term storage stability UV–visible spectra at room temperature and 4°C in water matrix: (*d*,*g*) AuNS-PVP-GalDH, (*e,h*) AuNS-PVP-AR, (*f*,*i*) AuNS-PVP-GalOx.

An assessment of the matrix and short-term storage stability of the AuNS bioconjugates was required prior to their use in colorimetric assays, the results of which are presented in figure 3. The AuNS bioconjugates were stable in all matrix environments with a marginal spectral blue shift while maintaining the characteristic AuNS profiles (figure 3*a–c*). Likewise, the AuNS bioconjugates were stable under short-term storage stability conditions for 96 h, and overall, storage at 4°C was optimal (figure 3*d–i*). These results show that AuNS bioconjugates exhibit adequate stability in a variety of matrices, as well as good short-term storage stability in water which was considered to be sufficient for use in the redox-mediated colorimetric assays that follow.

**Figure 4.**
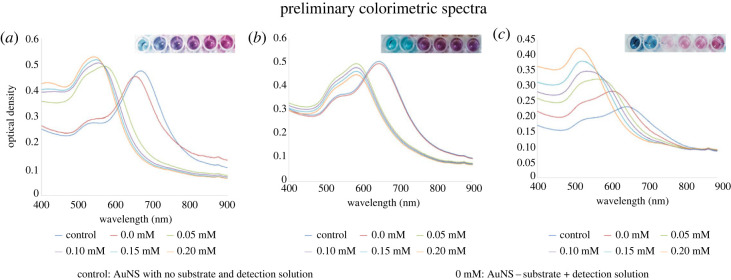
Preliminary colorimetric UV–visible profiles for AuNS-PVP in the presence of (*a*) NAD+, (*b*) NADPH and (*c*) H_2_O_2_.

Figure 4 depicts the preliminary colorimetric assays illustrating a direct correlation between NAD(P)/NAD(P)H and H_2_O_2_ concentrations and AuNS absorbance. The UV–visible spectral readings and colorimetric signals show that NAD(P)/NAD(P)H and H_2_O_2_ cause concentration-dependent changes in the AuNS absorption pattern, which most likely correlates to the nanostar transition from an anisotropic to quasi-spherical shape.

Thermodynamics predicts that NAD(P)+ and H_2_O_2_ will spontaneously react with Au. Using standard reduction potentials and half-cell reactions, we have theoretically described how NAD+ and H_2_O_2_ induce colorimetric and morphological transformations in AuNS. The standard redox potentials for the NAD(P)+/NAD(P)H and AuCl_4_^−^/Au pairs are −0.32 V, −0.324 V and +1.002 V, respectively [45–47].

4.1 $$\mathrm{Au}\to {\mathrm{Au}}^{3+}+3e^{-}\;E^{\circ }=-1.002\;\;V,$$

4.2 $${\mathrm{NAD}}^{+}+H^{+}+2e^{-}\to \;\mathrm{NADH}\;E^{\circ }=-0.32\;V,$$

4.3 $$\mathrm{NADPH}\to {\mathrm{NADP}}^{+}+2e^{-}+H^{+}\;E^{\circ }=+0.324\;V,$$

4.4 $$H_{2}O_{2}+2H^{+}+2e^{-}\to 2H_{2}O\;E^{\circ }=+1.78\;V,$$

4.5 $$2\mathrm{Au}+3{\mathrm{NAD}}^{+}+3H^{+}↔2{\mathrm{Au}}^{3+}+3\mathrm{NADH}\;E^{\circ }=+0.682\;\;V;\Delta G^{\circ }=-394.8\;\;\mathrm{kJ}$$

4.6 $$\mathrm{and}\;2\mathrm{Au}+3H_{2}O_{2}+6H^{+}↔2{\mathrm{Au}}^{3+}+\;6H_{2}O\;\;E^{\circ }=+2.782\;V;\Delta G^{\circ }=-1611\;\mathrm{kJ}.$$

The redox reactions are thermodynamically feasible and spontaneous, suggesting that NAD+ and H_2_O_2_ can induce morphological changes in AuNS. By contrast, the Au-NADPH redox reaction is not thermodynamically feasible; however, Huang *et al.* [48] proposed that in the presence of oxygen, Au nanoparticles can react with NAD(P)H, causing morphological changes visible spectrophotometrically [48]. By contrast, Hietzschold *et al.* [49] and Xiao *et al.* [45] proposed that NAD(P)H can facilitate the growth of silver and gold nanoparticles respectively in the presence of AgNO_3_ and AuCl_4_^−^ in the presence of reducing agents [49].

**Figure 5.**
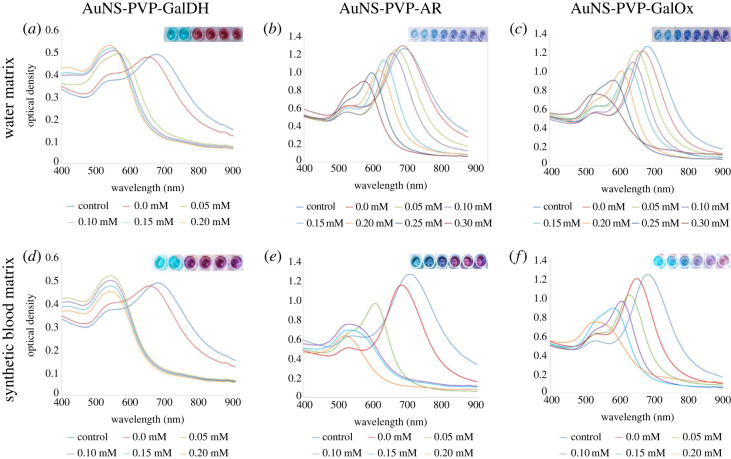
AuNS bioconjugate colorimetric spectra in water and synthetic blood with increasing galactose: (*a*,*d*) AuNS-PVP-GalDH, (*b*,*e*) AuNS-PVP-AR, (*c*,*f*) AuNS-PVP-GalOx.

**Figure 6.**
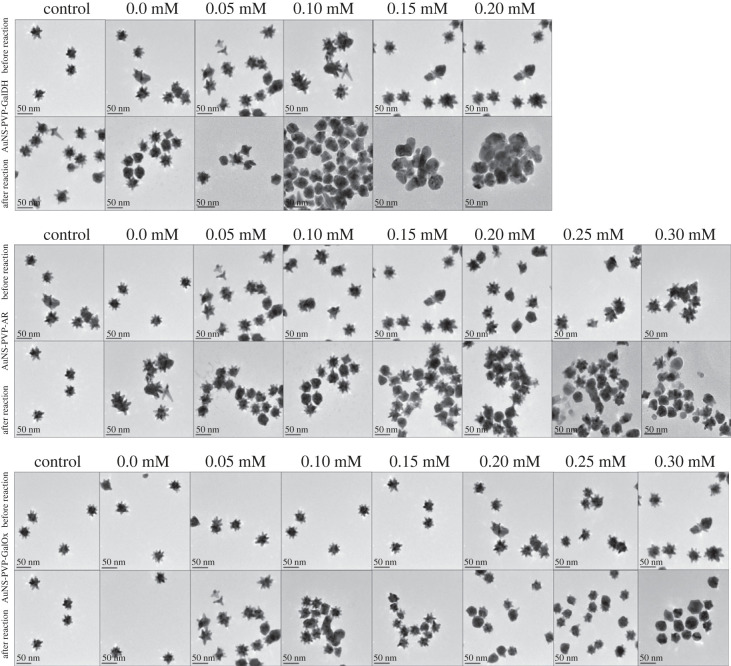
AuNS bioconjugate TEM images before and after colorimetric reactions in the water matrix.

Figures 5 and 6 depict the colorimetric assay results of AuNS bioconjugates in water and synthetic blood matrix. In both cases, increasing galactose concentrations cause a blue shift of varying magnitudes, followed by the transition of AuNS into quasi-spherical nanoparticles as shown in the UV–visible spectra profiles and TEM images in the water matrix.

The AuNS-PVP-AR assay required a 5 min incubation, whereas the AuNS-PVP-GalOx assay required a 1 min incubation to generate plausible colorimetric signals after adding the detection solution. By contrast, after adding the detection solution in all matrices, the AuNS-PVP-GalDH assay showed an instantaneous change to a wine-red colour. The visually observed colour changes, UV–visible spectral profiles and HR-TEM images suggest that the AuNS-PVP-GalDH assay has a narrow quantification range in all matrices, in comparison to broader ranges observed for the AuNS-PVP-AR and AuNS-PVP-GalOx assays.

Interestingly, quantitative ICP-MS data (table 1) before and after the reaction reveal that the quantities of Ag and Au remained nearly constant while the quantity of Na increased. This suggests that contrary to previous studies, NaOH participates in our colorimetric assays and that the quasi-spherical gold nanoparticles are not silver-plated, although the presence of Na in the lattice was not confirmed [50,51]. Overall, the AuNS-PVP-GalDH assay has the potential to be used qualitatively, while the AuNS-PVP-AR and AuNS-PVP-GalOx assays could be used semi-quantitatively.

**Table 1.** AuNS bioconjugate quantitative ICP-MS data before and after colorimetric reactions in the water matrix.

elements	AuNS-PVP-GalDH before reaction (ppm)	AuNS-PVP-GalDH after reaction (ppm)	**fold change**
23 Na	4.6	37.5	8.24
107 Ag	16.9	17.3	1.02
197 Au	39.1	39.8	1.02
elements	AuNS-PVP-AR before reaction (ppm)	AuNS-PVP-AR after reaction (ppm)	**fold change**
23 Na	4.2	37.5	8.96
107 Ag	16.4	17.3	1.05
197 Au	40.2	39.8	0.99
elements	AuNS-PVP-GalOx before reaction (ppm)	AuNS-PVP-GalOx after reaction (ppm)	**fold change**
23 Na	4.4	37.5	8.61
107 Ag	16.3	17.3	1.06
197 Au	39.7	39.8	1.00

**Figure 7.**
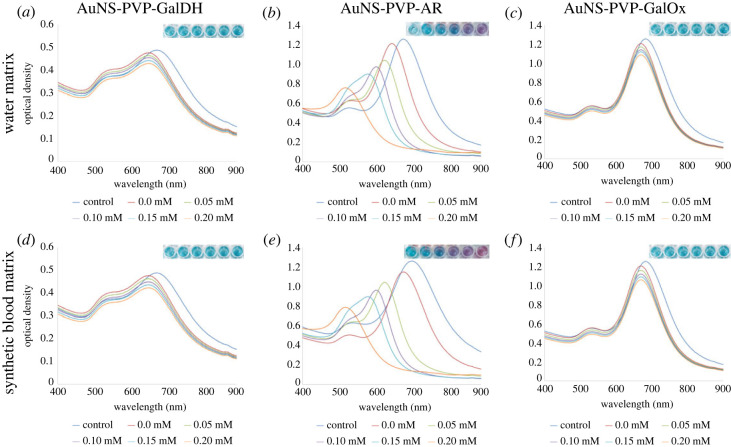
AuNS bioassay analyte specificity test. Glucose was used in place of galactose. (*a,d*) AuNS-PVP-GalDH, (*b,e*) AuNS-PVP-AR, (*c,f*) AuNS-PVP-GalOx.

GalDH and GalOx are galactose specific, whereas AR has a broad specificity for various aldoses and a high *K*_m_ for glucose and galactose [52–55]. Nevertheless, gold nanoparticles can act as a catalyst and can oxidize glucose [56–58]. It was therefore necessary to test the specificity of the assays and in particular the possible interference of glucose. The AuNS-PVP-AR assay UV–Vis spectra are blue-shifted in a concentration-dependent manner with increased glucose concentration, changing the nanostar morphology to quasi-spherical particles in water and synthetic blood matrices (figure 7*b*,*e*). By contrast, when exposed to glucose, the AuNS-PVP-GalDH and AuNS-PVP-GalOx assays showed no reaction or colour changes, indicating the usual blue shift in the spectrum induced by the detection solution and that the assays were galactose specific as expected (figure 7*a*,*c*) [52,53].

These results show that the proposed catalytic activity of the gold nanoparticles does not change the specificity of the AuNS-PVP-AR bioassay.

## 5. Discussion

Anisotropic AuNS were synthesized using a HEPES-mediated seedless one-pot synthesis procedure. HEPES acted as a reducing, shape-directing and stabilizing agent in this technique, resulting in heteromorphic nanostars of various diameters (figure 1*a*) [59,60]. Numerous studies have revealed that HEPES has a strong affinity for gold, which could be attributable to the sulfonate groups in its chemical structure [59,61,62]. This could explain its existence in the AuNS lattice post-synthesis, as evidenced by elemental analyses (figure 1*a*,*b*) and NMR analyses (figure 2*b*,*f*,*j*), both of which are consistent with prior studies. The anisotropic AuNS synthesized were characterized by the combination of two distinct UV–visible absorption peaks, which corresponded to the transverse plasmon resonance mode (plasmonic solid core) and the longitudinal plasmon resonance (plasmonic protuberant spikes) [44].

GalDH and AR are dimeric apoenzymes with a (*β*/*α*)_8_ barrel that allows NAD(P)+/NAD(P)H to bind and form apoenzyme–coenzyme complexes [63–65]. NAD(P)+/NAD(P)H operate as conformation primers, stabilizing the apoenzyme–coenzyme complex oligomeric catalytically active structure [65,66]. The significance of NAD(P)+/NAD(P)H coenzymes in the structure and activity of apoenzymes encouraged us to co-immobilize the apoenzyme–coenzyme complexes to the AuNS during the AuNS bioassay synthesis. Furthermore, as shown in figures 1 and 2, PVP capping caused slight modifications to the AuNS, the nanostar lattice structure was predominantly composed of gold and HEPES, and enzymes were successfully immobilized to the nanostars.

The bioassays' analyte plasmonic colorimetric sensing efficacy was investigated. The AuNS-PVP-GalDH assay produced an instantaneous colorimetric signal. By contrast, the AuNS-PVP-AR and AuNS-PVP-GalOx assays produced nearly comparable spectrum changes in water and synthetic whole blood matrices (figure 5). NADH, the reaction product of the AuNS-PVP-GalDH assay, is a robust competitive inhibitor of GalDH and also inhibits AuNS dissolution; this could explain the development of an instantaneous colorimetric signal, which is consistent with Liang *et al.*'s findings [67,68]. The assays, however, were based on the blue-shifted absorption band of nanostar (690 nm) to quasi-spherical nanoparticles (540 nm) upon NAD(P)+/NADPH and H_2_O_2_ biocatalytic reactions (figure 5). When employing synthetic blood, the bioassays had a potential drawback because the bulk of blood components have substantial absorption in the same range as the AuNS (figure 5*d–f*) [69–71]. Nonetheless, AuNS are highly sensitive, with an extinction coefficient greater than that of blood components; hence matrix dilution and a blank subtraction procedure were applied to facilitate result interpretation and analysis [72,73].

The transformation in size, shape and dielectric environment of the AuNS bioassays resulted in plausible plasmon band shifts and colorimetric signals (figure 5). Colorimetric signal generation in our bioassays could be pH-driven, enzyme-mediated or analyte-mediated, resulting in spectrum shifts and colorimetric changes [43]. Overall, the lack of change in Ag or Au concentrations in ICP-MS data (table 1) indicates no silver or residual gold cation reduction in the solution. As a result, we propose that either the remodelling phenomena or a typical ion exchange caused nanoparticle surface oxidation, followed by NAD(P)/NAD(P)H-mediated redox of Au^3+^ to Au^0^, with morphological and colorimetric changes to the AuNS.

Several enzymatic and non-enzymatic techniques for detecting NAD(P)+/NAD(P)H have recently been developed and published in peer-reviewed journals. These are primarily colorimetric and electrochemical detections, with some successfully integrated into miniaturized devices. Based on the dissolution of gold nanoparticles, Liang *et al.* [67] developed a high-sensitivity paper-based device for the rapid visualization of NADH [67]. Jafari *et al.* [74] recently suggested a colorimetric paper-based biosensor based on the phenylalanine dehydrogenase (PDH) enzyme for the highly sensitive and selective measurement of phenylalanine (Phe), employing cationic dyes as colorimetric mediators [74]. Messina *et al.* [75] devised a highly sensitive colorimetric method for the enzymatic detection of Phe in the presence of neo-formed NADH using tris(bipyridine) ruthenium(II/III) mediator [75]. Furthermore, Maugeri *et al.* [76] established a novel photothermal-contrast approach for Phe detection in human blood using PDH, resulting in the creation of *in situ* gold nanostructures in the presence of neo-formed NADH and AuCl_4_^−^ [76].

Our findings support Liang *et al*.'s [67] findings that neo-formed NADH inhibits AuNS dissolution. In our bioassays, the AuNS operate as colorimetric and sensing signal amplifiers [77], avoiding the need for dyes, in contrast to the biosensor designs proposed by Messina *et al.* [75] and Jafari *et al.* [74]. Furthermore, our understanding of redox chemistry (equation (4.5)) confirms Maugeri *et al*.'s [76] findings that neo-formed NADH can react with Au^3+^ to generate gold nanoparticles.

## 6. Conclusion

In conclusion, we report rapid and sensitive redox-driven galactose bioassays using NAD(P)/NAD(P)H and H_2_O_2_ for tailoring AuNS morphology and plasmonic properties. A plausible finding from the AuNS bioassays shows that in the absence of oxidase enzymes, dehydrogenase or reductase enzymes can be used instead, and NAD(P)/NAD(P)H can be used as redox agents in enzymatic plasmonic AuNS assays for analyte(s) quantification. We demonstrated that the AuNS-PVP-AR and AuNS-PVP-GalOx assays were semi-quantitative, whereas the AuNS-PVP-GalDH assay was a qualitative galactose assay. Overall, the enzyme-guided etching of the AuNS detected low concentrations of analytes, as shown in figures 5 and 7, and was supported by UV–visible spectrum profiles, TEM images, colorimetric data and ICP-MS data. With suitable assay tailoring, NAD(P)/NAD(P)H-dependent enzymes can be used to change AuNS morphology and plasmonic properties in complex matrices for analyte(s) detection. Thus, AuNS bioassays hold great promise for application in biomedical diagnostics for resource-constrained areas.

## Data Availability

The supporting data are accessible on the Figshare repository: https://figshare.com/s/d3c22f5d7c41c82f8d41.
